# 3D myocardial perfusion-CMR using a multi-transmit coil and k-t PCA reconstruction to detect flow limiting coronary stenosis

**DOI:** 10.1186/1532-429X-13-S1-P12

**Published:** 2011-02-02

**Authors:** Roy Jogiya, Amedeo Chiribiri, Andreas Schuster, Christian Jansen, Kalpa De Silva, Divaka Perera, Simon Redwood, Eike Nagel, Sebastian Kozerke, Sven Plein

**Affiliations:** 1 Kings College London, London, UK

## Purpose

To explore the clinical feasibility of 3D *k-t* PCA myocardial perfusion CMR on a multi-transmit 3T system in patients listed for invasive intracoronary pressure wire assessment.

## Background

Three-dimensional acquisition methods facilitated by undersampling in space and/or time have been proposed to overcome the limited cardiac coverage of dynamic first pass myocardial perfusion-CMR. Recently, temporally constrained k-t BLAST reconstruction using principal component analysis (k-t PCA) has been proposed to further improve temporal resolution of k-t undersampled data. In addition, multi-transmit technology has become available, which aims to improve image uniformity and SAR limitations at higher field strength. We have developed a pulse sequence for 3D perfusion-CMR that utilises both k-t PCA and multi-transmit technology at 3 Tesla.

## Methods

8 patients with suspected coronary artery disease underwent 3D perfusion MR imaging at rest and adenosine stress prior to angiography and fractional flow reserve assessment. *k-t* PCA accelerated perfusion CMR was performed on a 3T Philips Achieva Multi-transmit system (3D dynamic spoiled gradient echo, TR 2.0 ms, TE 1.0 ms, flip angle 30, matrix 160x160x80 mm, 16 slices of 5 mm thickness). FFR was measured in all vessels with visually significant stenosis using a pressure sensor-tipped wire (Volcano^®^). FFR < 0.75 was considered haemodynamically significant. Two experienced observers blinded to the results of the angiogram visually interpreted ischemia on CMR data as relative underperfusion of a sector within a slice or relative endocardial vs epicardial underperfusion within a coronary teritory. The performance of visual analysis of CMR to detect flow-limiting coronary stenosis on angiography was determined.

## Results

**Figure 1:**
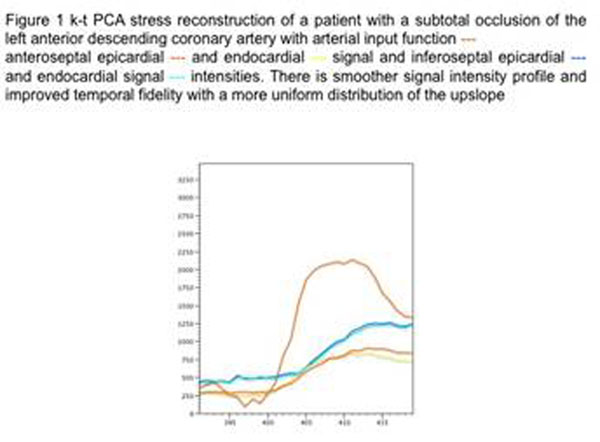


**Figure 2:**
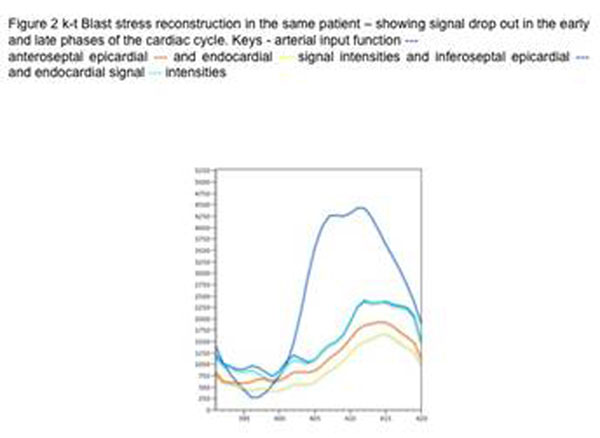


Acquisition was feasible in all patients. Signal intensity (SI) profiles showed improved temporal fidelity of k-t PCA (Figure 1) compared with standard k-t BLAST (figure 2) reconstruction SI profiles were similar across myocardial segments. Using k-t PCA Image quality was scored as “good” in 6 of the 8 cases. Perfusion deficits were seen in 4 of the 8 scans. Abnormal FFR was found in 4 patients with correlation between CMR and FFR in 6 of the 8 cases.

## Conclusion

3D myocardial perfusion imaging using Multi-transmit technology and k-t PCA reconstruction is technically feasible. Improved temporal fidelity was observed using multi-transmit technology. Perfusion deficits in patients with suspected CAD correlate well with invasive FFR measurements in this small pilot study.

